# Guidelines to support newly qualified professional nurses for effective clinical practice

**DOI:** 10.4102/curationis.v47i1.2527

**Published:** 2024-03-26

**Authors:** Warriodene Hansen, Sibusiso M. Zuma

**Affiliations:** 1 Department of Health Studies, Faculty of Human Sciences, University of South Africa, Pretoria, South Africa

**Keywords:** clinical environment, guidance, model development, newly qualified professional nurse, preceptor, preceptorship, support, transition

## Abstract

**Background:**

Transitioning to a professional role is difficult for newly qualified professional nurses. Given the challenges that these nurses experience during the transition to practice, support is essential for them to become efficient, safe, confident, and competent in their professional roles.

**Objectives:**

The purpose of this study was to explore the transition experiences of newly qualified professional nurses to develop a preceptorship model.

**Method:**

This study employed a qualitative approach to purposively collect data. Concept analyses were conducted applying the steps suggested by Walker and Avant, and the related concepts were classified utilising the survey list of Dickoff, James and Wiedenbach’s practice theory.

**Results:**

A preceptorship model for the facilitation of guidance and support in the clinical area for newly qualified professional nurses was developed. The model consists of six components, namely, the clinical environment, the operational manager and preceptor, the newly qualified professional nurse, the preceptorship, the assessment of learning, and the outcome.

**Conclusion:**

The study revealed that newly qualified professional nurses face many transition challenges when entering clinical practice. They are thrown far in, experience a reality shock, and are not ready to start performing their professional role. The participants agreed that guidance and support are needed for their independent practice role.

**Contribution:**

The preceptorship model for newly qualified professional nurses would be necessary for the transition period within hospitals. This preceptorship model may be implemented by nursing education institutions as part of their curriculum to prepare pre-qualifying students for the professional role.

## Introduction

Newly qualified nurses will not come to workplace knowing everything (Lewis & McGowan 2015:43). This change from student nurse to registered nurse is distressing (Sonmez & Yildirim 2016:104). During this period, newly qualified professional nurses find it difficult when applying recently learnt knowledge and skills (Blegen et al. 2015:642). The challenges with professional role transition are assigned to various factors, among others, such as needing more preparation, finding it difficult to bridge the theory-practice gap, and having no guidance or support (Canizares et al. 2015:176). In addition, Patterson, Boyd and Mnatzaganian (2017:103) found that the initial phase is critical to newly qualified professional nurses because of elevated stress levels, lack of theory-practice integration, and the risk of mistakes occurring.

Given the problems faced by newly qualified professional nurses experience during the transition to the independent practice role, support is essential for them to become efficient, safe, confident and competent in their professional roles (Lewis & McGowan 2015:43; Salmond & Echevarria 2017:16). According to Aldosari, Pryjmachuk and Cooke (2021:13), transition interventions are essential for newly qualified professional nurses to gain confidence. While preceptorship is identified as a critical need to improve clinical experience of newly qualified nurses (Freeling, Parker & Breade 2017:27). Hence, this study aimed to investigate the transition experience of newly qualified professional nurses towards professional nursing to develop a preceptorship model for newly qualified professional nurses during transition.

## Problem statement

The transition process to nursing is stressful, leaving newly qualified professional nurses feeling unprepared (Darawad, Mansour & Al-Niarat 2020:419). According to AlMekkawi and El Khalil (2020:315), newly qualified professional nurses seek support because of a gap in clinical training experiences. However, Woo and Newman (2020:86) found that newly qualified professional nurses do not feel overwhelmed but part of the team when they receive support from their supervisor and colleagues. During the course of this study the researcher found that the newly qualified professional nurses described that they felt protected by their student status when it came to working by themselves. A new workplace was overwhelming but receiving help from colleagues eased settling into their new workplace (Hansen, 2021:8). Furthermore, the researcher found that newly qualified professional nurses were dependent on guidance and support, but they found ways to perform their role effectively. Consequently, using Google and returning to their textbooks for demonstrations and information.

Significantly, the results of this study revealed that the newly qualified professional nurses needed support with clinical skills. As the researcher found during this study that the newly qualified professional nurses found their transition challenging when applying theory to practice, but they were eager to become independent. According to Hansen (2021:8), newly qualified professional nurses will be motivated if they were to be introduced to a programme to ease their transition period.

## Purpose of the study

The purpose of this study was to investigate the transition experiences of newly qualified professional nurses in order to develop a preceptorship model for them.

## Research methods and design

The research approach for this study was qualitative with a descriptive phenomenology design. The research approach was appropriate because the researcher explored the transition experiences of newly qualified professional nurses towards professional nursing to develop a preceptorship model. The research design was employed in this study to get accurate and detailed descriptions of the newly qualified professional nurses’ transition experiences. Furthermore, this approach was utilised in this study consisting of an empirical phase, where data were collected, a concept analysis phase, and the development of the model.

### Phase 1: Data collection

In this research study, semi-structured interviews were utilised to collect data. The researcher used a self-developed interview guide that was created for this study. Data were collected over a period of 6 months. The key informers in this study were newly qualified professional nurses who shared their transition experiences, preceptors and operational managers, who shared their perceptions about the newly qualified professional nurses’ transition and readiness for the independent practice role. Interviews were conducted with 25 participants who were purposively selected. The sample size was determined by data saturation. However, participants were selected based on an inclusion and exclusion criteria. The sample selected were 11 newly qualified professional nurses, 7 preceptors, and 7 operational managers.

### Phase 2: Concept analysis

The researcher utilised the steps suggested by Walker and Avant (2013:165) for concept analysis. The related concepts were classified utilising Dickoff, James and Wiedenbach’s (1968:434).

### Phase 3: Development of the model

The model was developed based on Chinn and Kramer’s (2018) elements, purpose, assumptions on which the model is based, definitions of the main concept, nature of the structure of the model, nature of the process of the model and relationship statements.

### Phase 4: Evaluation of the developed model

The model was evaluated by utilising the critical reflective questions suggested by Chinn and Kramer (2018). The model was shared with two nursing managers, six preceptors, and four newly qualified professional nurses from both research settings, to test for clarity, simplicity, generalisability, accessibility and importance of the proposed model applying Chinn and Kramer (2018:203)’s critical reflection questions.

### Enhancing trustworthiness

The trustworthiness of the data obtained was measured against the four constructs, namely credibility, transferability, dependability and conformability (Lincoln & Guba 1985:301). Table 1 depicts how the researcher applied these strategies.

**TABLE 1:** Trustworthiness methods.

Strategy	Description	
Credibility	Prolonged engagement	Just more than 6 months were spent collecting data and analysing data to ensure deep understanding of participants’ experiences.
	Member checks	Returned to participants to let them read transcripts and verify if the words match what they intended to say. The researcher emailed the transcripts to the participants that was not available.
	Triangulation	Multiple data sources, namely, newly qualified professional nurses, preceptors and operational managers were the informants in this study. Multiple theories were also utilised for model development.
Transferability	Thick description	Comprehensive description of research settings, participants and methods followed to conduct the study. Wrote a report to describe results.
Dependability	Audit trail	The researcher safeguarded all audio tapes during data collection, data transcripts and interview schedules, field notes, and notes about the research procedures.
Confirmability	The researcher kept an audit trail, kept raw data, kept a reflective journal, and gave thick descriptions of the findings.	

### Ethical considerations

This study was conducted in accordance with the Helsinki Declaration as revised in 2013. An application for full ethical approval was made to the Higher Degrees Committee of the Department of Health at University of South Africa (Unisa), and ethics consent was received on 21 March 2021 (ethics approval number Rec-2408 16-052). Written permission from the Western Cape Department of Health and the chief executive officers of the two hospitals was obtained. To participate in this study, the participants met the inclusion criteria. Potential participants received letters containing the purpose of the study and how they would be involved in the study. The researcher formulated a written consent form which the participants signed after receiving information about the research. Participation was out of free will. The identity of the hospital was redacted and that of the participants were kept confidential using codes. Data collected were saved on a password-protected laptop.

## Results and discussion

The results show the significance of support and guidance for newly qualified professional nurses. The results were used as the basis for the development of the preceptorship model for newly qualified professional nurses.

### Phase 1: Data collection

The participants were purposefully selected to get rich information about the professional transition of newly qualified professional nurses. To achieve the purpose, the researcher approached participants that experienced transition to the professional role of nursing and that have preceptor experience.

This study was conducted in the Western Cape Province in the Republic of South Africa. This study was conducted at two research settings. The newly qualified nurses appointed at these hospitals are from the nursing colleges and universities located in the Western Cape province. They either obtained diplomas or degrees. The phenomenon of interest in this study were the participants’ experiences and challenges, the transition of newly qualified professional nurses, preceptors and operational managers involved in a preceptorship based on their realities. All the interviews were conducted in these two settings.

The researcher conducted interviews one participant at a time and recorded the interviews with the participants’ consent. An interview guide was utilised, however the participants had freedom to share their lived experiences. One central research question was asked per participant group: How would you describe the professional transition of newly qualified professional nurses? The questions were from the researcher’s experience as a preceptor for data collection during interviews. The questions were open-ended with probes, allowing the participants to respond in their own words. The interviews were conducted face-to-face and individually. This gave the researcher the opportunity to facilitate the interview.

The researcher used these notes to reflect what happened during the interview for bracketing and complement the audio recording of the participants. Initial scrawls were made by the researcher during the interviews. The researcher used field notes to describe the participants’ actions and emotions observed during the interviews. The researcher also made preliminary jottings during the interviews.

### Phase 2: Concept analysis

The central concept was identified. Dictionary and theoretical definitions were provided to clarify the concept. In addition, the use of the concept in real-life situations is explained through model and contrary cases. Lastly, the related concepts were determined and classified.

#### Selection of the concept

The most significant finding of this study was that the participants observed the newly qualified professional nurses’ transitioning to the professional role challenging, as the newly qualified professional nurses felt not ready, and experienced a reality shock during the transition period. Furthermore, the operational managers agreed that newly qualified professional nurses will be motivated to learn if introduced with a clinical teaching and learning programme based on them as adult learners.

**FIGURE 1:**
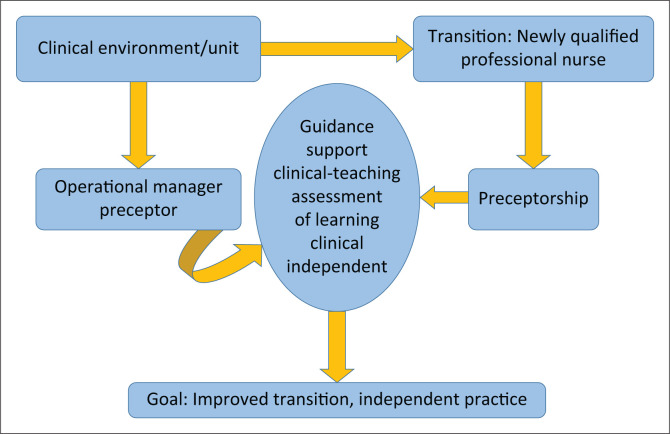
Concept map of preceptorship.

As a result, it was evident that the newly qualified professional nurses needed professional guidance and support; therefore, the concept that appeared in the themes and discussions was, preceptorship. Preceptorship was selected as the main concept to develop a model to facilitate guidance and support in the clinical area for newly qualified professional nurses. The results from the analysed data informed the identification, definition and classification of concepts related to preceptorship. Figure 1 presents a concept map, informed by the findings of this study.

#### Purpose of the analysis of the concept

In this study, it was important to understand the preceptorship concept to provide adequate guidance and support to newly qualified professional nurses. Moreover, it was important to determine what the development of a preceptorship model for newly qualified professional nurses meant to the transition of newly qualified professional nurses.

#### Identify uses of the concept

In this study, the concept of preceptorship will be used for the development of a preceptorship model for newly qualified professional nurses.

#### Definition of preceptorship

According to the Blackwell’s Nurses Dictionary (2010:313), preceptorship is a phase where guidance and support are provided by a preceptor for at least the first 4 months to newly qualified professional nurses.

#### Concept definition of preceptorship

The support during preceptorship can also be positive whereby it is used for coaching, mentoring and for facilitation methods that can identify learning needs, apply assessment skills, and can promote the implementation of best practice guidelines (Botma 2016:5). Although preceptorship is described as support given by a preceptor, it can also be an extension period of learning for newly qualified professional nurses (Jenkins, Oyebode, Bicknell, Webster, Bentham & Smythe 2021:2932). Arbabi, Johnson and Forgrave (2018:49) found that preceptorship is positive as it decreases medicine administration errors, improves quality of care delivered by newly qualified professional nurses, and increases nursing knowledge.

#### Derivation of preceptorship

In this study, the concept preceptorship means a period where the newly qualified professional nurse will be engaged in a guidance and support relationship with a preceptor. During this relationship, learning opportunities will be provided to increase clinical independence and clinical ability.

For the preceptorship to take place, newly qualified professional nurse must experience a transition period or be new to the professional role. The process of the preceptorship includes clinical teaching activities planned for adult learners. The clinical learning activities are based on objectives of the unit or department, to be achieved through the preceptorship. The process includes active experimentation of the clinical skills learned and making sense of the learning through demonstrations and having a dedicated preceptor. The goal of the preceptorship model is newly qualified professional nurses who are ready and prepared to fulfil the independent practice role.

#### Determine the defining attributes

The concept is the preceptorship of the newly qualified professional nursing within the clinical area. Determining the defining attributes is an attempt to identify attributes that are frequently associated with the concept, which will provide greater insight into the concept (Walker & Avant 2013:162). The defining attributes are summarised in Table 2.

**TABLE 2:** Defining attributes of support and guidance of preceptorship.

Attribute	Related attribute
Assistance	Helping through offering support
	Give advice
	Clinical teaching
	Directing
	Demonstration
	Provide information
Clinical output	Deliver high quality service to clients
Coaching	Provide learning opportunities
	Motivate
	Clinical independence
Competence	Assess learning activities
	Improved clinical ability
Independence	Increase the clinical ability and knowledge
	Self-directed
Colleagues	Create environment for peer support
	Be a mentor
	Be knowledgeable
	Have expertise
Leader	Influencer
	Facilitator of learning
Clinical teacher	Create opportunities to learn and develop
	Help a person
	Ease challenges
	Role model
	A resource to others
	Respected person
Mentoring	Guidance
	Support
	Advice

#### Identification of a model case and a contrary case

In this study. the researcher described constructed examples of facilitation of guidance and support by means of preceptorship. The researcher described a constructed model case to demonstrate the facilitation of guidance and support by means of preceptorship as well as a constructed contrary case.

#### Antecedents for preceptorship

For the concept to take place, there are vital elements for it to happen. The researcher identified this element as the preceptorship, which is the event to take place. The preceptorship was identified as the antecedent because the preceptorship model was developed for the clinical teaching of newly qualified professional nurses and through the preceptorship, guidance and support are given.

#### Consequences for preceptorship

An effective preceptorship may achieve the outcome and result in improved transition of newly qualified professional nurses towards the independent practice role. Therefore, many efforts are made by the agents to reach the outcome of the preceptorship model. All, to increase the newly qualified professional nurses’ readiness and preparedness for the independent practice role.

#### Concept classification

The researcher employed the survey list of Dickoff et al. (1968:450) to classify and identify the concepts related to the central idea. The survey list includes the agent, recipient, context, dynamic, process, and terminus. The researcher developed a reasoning map to create an understanding of classifying the concepts (see Table 3).

**TABLE 3:** The tabulated form of the concept classification according to the survey list.

Survey list	Description
Agent	**Who or what performs the activity?** *The operational managers and the preceptors.*
Recipients	**Who or what is the recipient of the activity?** *The newly qualified nurses.*
Context	**In what context is the activity performed?** *Preceptorship is performed in a clinical unit within a hospital setting.*
Process	**What is the process and/or technique of the activity?** *In this study, newly qualified nurses will be offered support and guidance to ease transition into the professional role; this will be offered through a preceptorship.*
Dynamics	**What is the energy source of the activity?** *Clinical ability, support and guidance and inherent nursing skills. The motivation for newly qualified nurses by the preceptors to improve competency towards better clinical practice.*
Terminus/outcome	**What is the endpoint of the activity?** *The outcome of the preceptorship is the readiness and preparedness of the newly qualified nurse’s independent clinical practice.*

### Phase 3: Development of the model

In this research study, the preceptorship model for the facilitation of guidance and support in the clinical area for newly qualified professional nurses will provide opportunities for the nurses through facilitation in order to increase their clinical ability. The model has been constructed from various theoretical frameworks and was followed by an evaluation of the model, applying Chinn and Kramer’s (2018:203) critical reflection questions.

#### Overview of the model

The model will serve as a framework of reference to facilitate the guidance and support for newly qualified professional nurses to develop their readiness, preparedness, and independence in clinical practice. This model is a schematic representation of how preceptorship of newly qualified professional nurses will prevent the challenges of transitioning, feeling not ready, and these nurses experiencing a reality shock. Based on the empirical data collected from the participants in this study, it was agreed that newly qualified professional nurses need support and guidance to be clinically competent and independent. The preceptorship model for the facilitation of guidance and support in the clinical area for newly qualified professional nurses has phases that lead to the outcome, readiness, and preparedness by the newly qualified professional nurse’s independent practice role.

#### Purpose of the model

The purpose of this model is to serve as a guide to mitigate the challenges and realities of transitioning newly qualified professional nurses experience within the clinical environment.

#### Context of the model

The context of the model is the clinical environment, where the newly qualified professional nurses were assigned to work in a unit in the hospital. For preceptorship to take place, the unit the newly qualified professional nurses work in must be a conducive environment. To have a conducive environment, the operational manager collaborates with the preceptor in planning and facilitating the preceptorship. The newly qualified professional nurses come to the clinical environment as novices to the independent practice role. The newly qualified professional nurses are expected to practise competently and independently, taking responsibility and accountability for actions. However, being unsure and unprepared for their role impacted their self-concept.

#### Assumptions of the model

According to Chinn and Kramer (2018:199), assumptions are the truths the researcher gives that hold crucial value to the theoretical reasoning of the model. The assumptions of the model have been developed by applying the following concepts and constructs from various theoretical frameworks obtained from reviewing scholarly literature. The survey list of Dickoff et al. (1968), Benner’s novice to expert theory (1982), King’s theory of goal attainment (1992), and Kolb’s learning cycle of experiential learning (1984) were applied to construct this model.

#### Definition of the concept

The selected concept was preceptorship. The concept was defined during the concept analysis.

#### Nature of the process description of the model

**The overall aim of the preceptorship model:** The overall aim of this model is to facilitate the effective preceptorship of newly qualified professional nurses. The model is a guide to preceptors and operational managers on how to facilitate preceptorship within their unit.

**Facilitative process:** The facilitative process refers to the planning and implementation of guidance and support of newly qualified professional nurses within the clinical area. This facilitation will be through preceptorship within the unit.

#### The interrelated phases of the constructive guidance and support phases

The main concepts in the model were guidance and support. The model’s related concepts were contextualised to give the main concepts essential meaning. The interrelated phases clarify the ideas on which the model was developed.

**Guidance and support:** In this study, newly qualified professional nurses experienced challenges with transitioning to their professional role. This caused them to experience a reality shock, feeling unprepared and not ready. The preceptors would use the preceptorship model for the facilitation of guidance and support in the clinical area during transition of newly qualified professional nurses.

**Interrelated phases:** The preceptorship model for the facilitation of guidance and support in the clinical area for newly qualified professional nurses has interrelated phases that assisted in the construction and description of the model.

#### The clinical environment

The clinical environment is the context, as described by Dickoff et al. (1968:422), where the facilitation of guidance and support takes place. On assignment to the clinical environment, the newly qualified professional nurses are novices and beginners (Benner 1982:402), with no minimal professional experience and with marginal acceptable clinical performance. The newly qualified professional nurses have the personal perception of themselves being not prepared and ready, and have a goal to improve this (King 1992:24). In this phase of the model, the newly qualified professional nurses experience the transition to nursing and their guidance and support are being facilitated.

**Clinical teaching and learning:** In this phase of the model, guidance and support are facilitated through interpersonal and social interaction (King 1992:24) with the operational manager, preceptor, and peers. Through the clinical teaching and learning, the newly qualified nurses gain concrete experience; they observe and can reflect and form abstract meanings of the clinical teaching and learning activities (Kolb 1984:21). This is also the process (Dickoff et al. 1968:422) of the facilitation of guidance and support by means of the preceptorship model.

**Assessment of learning:** In this phase, the newly qualified professional nurse’s orientation to learning is determined by evaluating competence, proficiency, and intuitive grasp (Benner 1982:402) of their clinical ability. The assessment of learning is the active experimentation as described by Kolb (1984:21) where the newly qualified professional nurses demonstrate the level of their clinical ability.

**Clinical independence:** At this phase, the outcome (Dickoff et al. 1968:422) and goal of the preceptorship, the readiness and preparedness of the newly qualified professional nurse is determined.

#### The nature of the structure of the model

**FIGURE 2:**
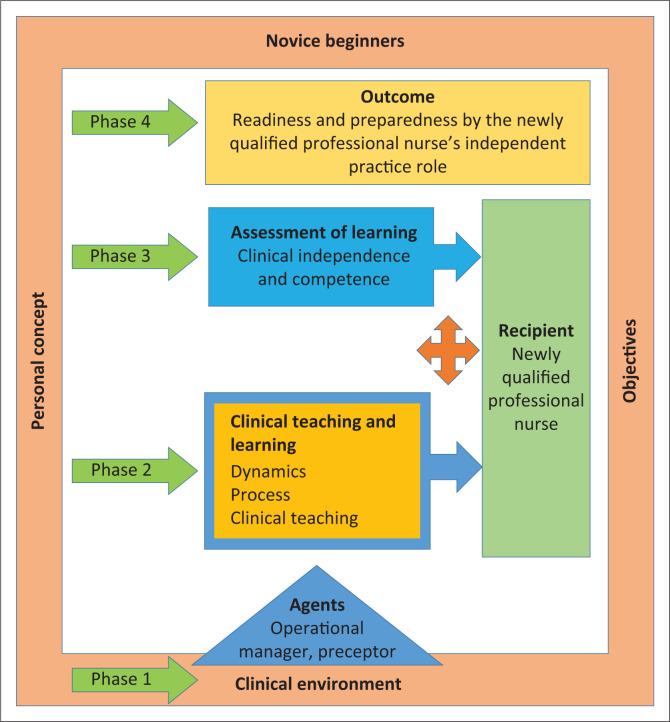
Preceptorship model for the facilitation of guidance and support in the clinical area for newly qualified professional nurses.

The structure of a model refers to the conceptual relation within it, and its description adds meaning to the concepts. Figure 2 illustrates the structure of the preceptorship model for the facilitation of guidance and support in the clinical area for newly qualified professional nurses.

The model is one structure that is multifaceted and contains six components:

- The context wherein the preceptorship will occur, that is the clinical environment, is represented by the peach rectangle frame. This the starting point of the preceptorship model when newly qualified professional nurses arrive as novices with limited experience. They have a personal goal. The preceptorship objectives are determined in this phase.
- The blue triangle pointing upwards represents the operational manager and preceptorship. They are vital to the facilitation of the preceptorship and influence all the phases within the preceptorship and influence the newly qualified professional nurse and outcome.
- The light blue rectangle with the arrow pointing to the right represents the assessment of the newly qualified professional nurse and it influences the outcome.
- The gold rectangle represents the outcome of the preceptorship model.
- The green arrows on the left represent the phases of the preceptorship model. The green rectangle shape to the right of the model represents the recipient.
- The orange four-way arrow demonstrates the newly qualified professional nurse not being a passive recipient but is actively participating in the phases of the preceptorship.

#### Relationship statements

According to Chinn and Kramer (2018:182), relationship statements interrelate the concepts of the model and they describe and predict the nature of interactions between concepts of the model. The following are the relationship statements created for this model:

- The clinical environment as the context of this model influences the preceptorship of newly qualified professional nurses. The newly qualified professional nurses are new to the independent practice role and lack the skills to function independently. This creates an opportunity for guidance and support through preceptorship. This is where the transition is experienced, and the preceptorship model implemented.
- The operational manager and preceptor as agents are vital to the implementation and sustaining of the preceptorship. The agents are important to the preceptorship model till the outcome of the preceptorship is reached.
- The newly qualified professional nurse is the recipient of this model. With their adult learning experiences, the newly qualified professional nurses are the focus of the preceptorship, and the dynamic processes of the preceptorship are built around them.
- The dynamics that drive the preceptorship of newly qualified professional nurses are support and guidance, clinical ability, and independence and inherent nursing skills.
- The procedure influences the outcome of the preceptorship of newly qualified professional nurses. The procedure involves learning outcomes, teaching activity, assessment of learning, and clinical independence and competence.
- The implementation and sustainability of the preceptorship model for newly qualified professional nurses depend on the agents and recipients, who all collaborate within the preceptorship.
- Support and guidance, clinical ability, and independence and inherent nursing skills are the elements that cause interaction between the agents and recipients. This will have an impact on the readiness and preparedness of the newly qualified professional nurses.

### Phase 4: Evaluation of the developed model

The model was shared with two nursing managers, six preceptors and four newly qualified professional nurses from both research settings, to test for clarity, simplicity, generalisability, accessibility and importance of the proposed model applying Chinn and Kramer (2018:203)’s critical reflection questions.

#### Is this model clear?

This model’s clarity was achieved by identifying and classifying the concepts. The researcher utilised the survey list of Dickoff et al. (1968) to give the model structural clarity. To ensure semantic clarity, the researcher defined the concepts of the model in order to give them empirical meaning. The researcher wanted to establish if the concepts were clearly defined and fit the relationships with the structure of the model.

#### Is the model simple?

Furthermore, Dickoff et al. (1968) state that complicated models have many relational components within the theory, and simple models have fewer relational components. This model is simple and easy to understand. The model’s components and elements are revealed and explained. The researcher explained the procedure that will facilitate the preceptorship as well as the dynamics which was based on the findings from the clinical settings. The researcher highlighted how these aspects will influence the newly qualified professional nurses’ readiness and preparedness for the professional role.

#### Is this model general?

This model was designed for newly qualified professional nurses experiencing professional transitioning but can be applied to any teaching and learning activity that requires teaching newly qualified staff. This model can also be utilised in undergraduate nursing where clinical support and guidance are of concern.

#### Is this model accessible?

The model will be published in academic journals, presented at nursing conferences, and made available to the clinical settings where the data were collected. In addition, the researcher will present the model to the nursing managers of the clinical settings where data were collected during workshops and meetings to explain its purpose.

#### Is this model important?

This model is important to all stakeholders striving to deliver a highly efficient service and quality to clients visiting their facilities. This model was the first created for the preceptorship of newly qualified professional nurses and to address the professional transition challenges highlighted in this study, namely reality shock and feeling not ready.

## Conclusion

In this article, the process of the development of this preceptorship model is described. This preceptorship model for newly qualified professional nurses will add information to the practice and nursing education. This model can be used in clinical settings during newly qualified professional nurses’ transition period and with any newly qualified person’s learning activity. By applying this model, newly qualified professional nurses may experience a positive transition period, improving their clinical competence. This study found a need for professional support and guidance of newly qualified professional nurses in order to help them prepare for their role in enhancing their clinical independence. The developed preceptorship model for the newly qualified professional nurses will positively contribute to the transition of newly qualified professional nurses, thus ensuring that they receive clinical training from trained preceptors and support from nursing managers who collaborate to achieve an effective preceptorship. Thus, the results of this study can greatly benefit the body of knowledge of nursing education and the nursing profession.

## Limitations

A limitation of this study is the restriction of the study to two hospitals within the same region and province, which limits the generalisation. A further limitation to generalisation is that this model employed a qualitative approach and used a non-probability sampling technique. It would be useful to evaluate the preceptorship model developed through this study at a clinical facility.

## Recommendations

### Recommendations for further research

It is recommended that a study be conducted to implement the concepts of the preceptorship model for newly qualified nurses in undergraduate nursing. The study should include higher education institutions, clinical institutions, and nursing students.

### Recommendations for nursing education

This model should be incorporated in the nursing curriculum and be utilised as a transition tool for students about to be newly qualified nurses.

### Recommendations for nursing practice

Implementing the preceptorship model for newly qualified nurses is advisable for all newly qualified nurses. Nursing management should attend workshops about the preceptorship model for newly qualified professional nurses.
